# Chronic kidney disease, health-related quality of life and their associated economic burden among a nationally representative sample of community dwelling adults in England

**DOI:** 10.1371/journal.pone.0207960

**Published:** 2018-11-26

**Authors:** Nga T. Q. Nguyen, Paul Cockwell, Alexander P. Maxwell, Matthew Griffin, Timothy O’Brien, Ciaran O’Neill

**Affiliations:** 1 Centre for Public Health, Queen's University Belfast, Belfast, United Kingdom; 2 University Hospitals Birmingham, Birmingham, United Kingdom; 3 National University of Ireland Galway, Galway, Ireland; Consiglio Nazionale delle Ricerche, ITALY

## Abstract

Chronic kidney disease (CKD) affects up to 15% of the adult population and is strongly associated with other non-communicable chronic diseases including diabetes. However, there is limited information on a population basis of the relationship between CKD and health-related quality of life (HRQoL) and the consequent economic cost. We investigated this relationship in a representative sample in England using the 2010 Health Survey for England. Multivariable Tobit models were used to examine the relationship between HRQoL and CKD severity. HRQoL was converted to quality adjusted life year (QALY) measures by combining decrements in quality of life with reductions in life expectancy associated with increased disease severity. QALYs were adjusted for discounting and monetised using the UK threshold for reimbursement of £30,000. The QALYs were then used in conjunction with forecasted prevalence to estimate the HRQoL burden associated with CKD among individuals with diabetes up to 2025. Individuals with more severe CKD had lower HRQoL compared to those with better kidney function. Compared to those with normal/low normal kidney function and stage 1 CKD, those with stage 2, stage 3 with albuminuria and stage 4/5 CKD experienced a decrement of 0.11, 0.18 and 0.28 in their utility index, respectively. Applying the UK reimbursement threshold for a QALY, the monetised lifetime burden of reduced HRQoL due to stage 2, stage 3 with albuminuria and stage 4/5 CKD were £103,734; £83,399; £125,335 in males and £143,582; £70,288; £203,804 in females, respectively. Utilizing the predicted prevalence of CKD among individuals with diabetes mellitus, the economic burden of CKD per million of individuals with diabetes is forecasted at approximately £11.4 billion in 2025. In conclusion, CKD has a strong adverse impact on HRQoL in multiple domains. The estimated economic burden of CKD among individuals with diabetes mellitus in the UK is projected to rise markedly over time.

## Introduction

Chronic kidney disease (CKD) has become a global public health issue whose economic impact has increased over time [1–3]. It is among the five most common causes of reduced life expectancy accounting for nearly one million deaths in 2013 [4]. In 2009–2010, the NHS in England spent approximately £1.45 billion (1.3% their budget) to cover direct and indirect CKD treatment costs [5], while in the USA, end-stage renal disease treatment costs alone amounted to $35 billion (6.3% of their annual expenditure) of the Medicare program [6]. The effects of CKD extend beyond healthcare costs and premature mortality. A systematic review of patient-reported outcome measures (PROMs) used in CKD supported the use of preference-based utility measures, favouring the EuroQoL EQ-5D [7]. Using this a number of studies have examined the relationship between health-related quality of life (HRQoL) and CKD demonstrating that those with more severe CKD experience reduced HRQoL [8–17].

While contributing to the literature these papers exhibit a number of limitations. First, as the impact on HRQoL might legitimately be expected to be greatest among those with more advanced CKD, many studies omit those with less advanced kidney disease [13–16] and therefore do not capture the full burden of CKD. Second, while several studies use multivariable analyses to control for covariates when estimating the impact of CKD on HRQoL, they adopt an ordinary least squares (OLS) approach that fails to account for the censored nature of the HRQoL outcome measure [8,10,11,13,14,17]. Consequently, these studies may produce biased estimates of the effect of CKD on HRQoL. Third, several studies include among their covariates some co-morbid conditions such as hypertension [8,11] and diabetes [8–13], conditions that may cause CKD and thus be endogenous to it. This may also result in biased estimates.

In this study, we examine the relationship between HRQoL and CKD among a representative sample of community dwelling individuals living in England that includes those with CKD at various levels of severity including none. We use a modelling approach that adjusts for the censored nature of HRQoL and compare models with and without potentially endogenous covariates. For illustrative purposes, we estimate the QALY decrement associated with CKD, value this in monetary terms and using forecasts of disease prevalence, estimate the HRQoL burden among patients with diabetes related to CKD in the UK up to 2025.

## Methods

Data were extracted from the Health Survey for England, an annual survey of community dwelling adults. Data from the 2010 survey which contained a specific module on measured kidney function and HRQoL were used [18]. For this study, eligible participants were the 2796 individuals aged over 16 who had a valid serum creatinine value on which CKD could be staged.

Kidney disease was staged by using the 4-variable MDRD estimated glomerular filtration rate (eGFR) equation and albuminuria quantified by a spot urinary albumin/creatinine ratio (ACR). Participants were categorised into one of seven groups: normal (eGFR >90 ml/min/1.73m^2^ and normal albuminuria), low normal (eGFR>60&<90 and normal albuminuria), stage 1 CKD (eGFR ≥90 and micro or macro albuminuria), stage 2 CKD (eGFR>60&<90 and micro or macro albuminuria), stage 3 CKD without albuminuria (eGFR >30&<60 and normal albuminuria), stage 3 CKD with albuminuria (eGFR >30&<60 and micro or macro albuminuria), and stage 4/5 CKD (eGFR<30 regardless of albuminuria). Micro albuminuria (higher urinary albumin excretion) and macro albuminuria (very high urinary albumin excretion) were based on an ACR of 30–300 mg/mmol and >300 mg/mmol respectively.

The EuroQoL EQ-5D-3L instrument was used to elicit self-reported health. Health states were described across five domains: mobility, self-care, usual activities, pain/discomfort and anxiety/depression at three levels of severity [18]. The *eq5d command* was used to convert self-reported health into a preference weight using the English value set [19].

Sociodemographic characteristics shown to impact on quality of life (QoL) among individuals with CKD were extracted for inclusion in the analysis. These included age [8,9,11,14,16], gender [8–11,13,16], religiosity [15,16], location [15], ethnicity [13,14], education level [8,16], marital status [8,16], and income [8]. In this study, sociodemographic variables comprised equivalised household income, age (less than 50 years old and 50 years old and older), gender (male and female), marital status (single and married/civil partnership), education (less than 19 years of education and 19 years or more of education), ethnicity (White British and other ethnicities), religion (no religion and have religion), location (urban area and rural area). Equivalised household income was calculated by dividing the total income of a household in the 12-month period preceding the survey (after tax and other deductions) by the converted number of household members. The household members were converted into equalised adults using the modified OECD (Organisation for Economic Co-operation and Development) equivalence scale [20]. Equivalised household income was then converted to Quintiles: Highest Quintile (>£45,138.89), Second highest Quintile (>£29,166.67 & ≤£45,138.89), Middle Quintile (>£19,090.91 & ≤£29,166.67), Second lowest Quintile (>£11,142.86 & ≤£19,090.91) and Lowest Quintile (≤£11,142.86).

### Data analysis

Participant characteristics were compared between different kidney disease status using χ^2^ tests for categorical variables. Descriptive statistics were used to describe kidney disease status among groups stratified by age, gender, socio-economic status, marital status and ethnicity.

A series of multivariable Tobit models in which kidney disease and sociodemographic characteristics were regressed on utility scores were estimated. The relationship between kidney disease and specific domains of health captured in the EQ-5D were analysed using ordered Probit models controlling for the same covariates.

As hypertension and diabetes are the two most common comorbidities associated with CKD [8–13], we conducted sensitivity analyses in which these were included as well as omitted as regressors. We also estimated a saturated model that included all identified health conditions in HSE 2010 (including: neoplasms, endocrine and metabolic, mental disorders, nervous system, eye complaints, ear complaints, heart and circulatory system, respiratory system, digestive system, genitourinary system, skin complaints, musculoskeletal system, infectious disease and blood and related organs issue) as covariates.

We weighted the sample for nurse-based measures to account for representativeness. This weighted tool was provided by HSE 2010, taking into account the non-response to nurse section from respondents. All P-values were 2-sided with alpha = 0.05 as the threshold for statistical significance.

### Projected burden

The median age of each CKD group was estimated from the data for males and females. Life expectancy by age group and gender were based on Turin et al [21]. As HRQoL might be expected to deteriorate with age, the decrement in utility related to CKD was adjusted to take account of each CKD age group using Ara et al [22]. The QALY decrement associated with CKD was based on the reduction in life expectancy and reduction in HRQoL between those with CKD at a given stage and those without. A discount rate of 3.5% consistent with that recommended by NICE [23] was applied to years experienced in the future and the result was monetised using the NICE threshold willingness to pay for a QALY of £30,000. The monetised value of QALYs loss was extrapolated using estimates for the prevalence of CKD by stage among individuals with diabetes to forecast the HRQoL loss in monetary terms for CKD in a population with diabetes up to 2025 [24]. Full details are set out in the supplementary file.

## Results

### Descriptive statistic

The characteristics of respondents are shown in Table 1. The Normal and Low normal categories were combined to Normal/Low normal GFR group in the interests of parsimony while allowing for the comparison between normal kidney function and other CKD levels. The mean age of the normal/low normal GFR group was 48.7 years old (±15.9). The mean age of participants increased with the severity of CKD, from 44.6 (±18.2) in stage 1 CKD to 74.1 (±13.4) in stage 3 CKD with albuminuria. A majority of the respondents were female, with the exception of stage 3 CKD with albuminuria where there were more males (60%) than females (40%). Most people in the normal/low normal eGFR groups were in the highest quintiles of income, the majority of respondents with CKD at other stages were in the lower income quintiles. Most participants were married, White British, living in an urban area and having no religion. Except for stage 4/5 CKD, the education level decreased with the severity of CKD, from 28% having more than 19 years of education in normal/low normal kidney function group to 8.6% of those in stage 3 CKD with albuminuria. Regarding comorbidities, one fifth of patients with stage 3 CKD and albuminuria had diabetes and two thirds of those with stage 4/5 CKD had diabetes. The proportion of respondents with hypertension increased with the severity of CKD, ranging from 28.4% (normal/low normal kidney function) to 100% (stage 4/5). In general, the older, less well-educated and poorer respondents were more likely to have reduced kidney function.

**Table 1:** Demographic characteristics, socioeconomic status and comorbidities of participants stratified to eGFR level.

	Normal/Low normal GFR n = 2,439		Stage 1 CKD n = 56		Stage 2 CKD n = 106		Stage 3 CKD (Without albuminuria) n = 155		Stage 3 CKD (With albuminuria) n = 35		Stage 4/5 CKD n = 5		P-value
	N	%	N	%	N	%	N	%	N	%	N	%	
**Age (Mean, SD)**	48.7	15.9	44.6	18.2	60.0	17.4	65.3	14.8	74.1	13.4	72.2	10.3	p < 0.001
**Age**													p < 0.001
< 50 years old	1,298	53.2%	33	58.9%	30	28.3%	26	16.8%	3	8.6%	0	0.0%	
≥ 50 years old	1,141	46.8%	23	41.1%	76	71.7%	129	83.2%	32	91.4%	5	100.0%	
**Gender**													P = 0.003
Male	1,084	44.4%	19	33.9%	53	50.0%	72	46.5%	21	60.0%	2	40.0%	
Female	1,355	55.6%	37	66.1%	53	50.0%	83	53.6%	14	40.0%	3	60.0%	
**Income**													p < 0.001
≤ £11,142.86	331	13.6%	16	28.6%	22	20.8%	21	13.6%	5	14.3%	1	20.0%	
>£11,142.86 ≤£19,090.91	406	16.7%	7	12.5%	17	16.0%	29	18.7%	11	31.4%	0	0.0%	
>£19,090.91 ≤£29,166.67	504	20.7%	14	25.0%	27	25.5%	50	32.3%	10	28.6%	2	40.0%	
>£29,166.67 ≤£45,138.89	572	23.5%	11	19.6%	25	23.6%	28	18.1%	7	20.0%	2	40.0%	
>£45,138.89	626	25.7%	8	14.3%	15	14.2%	27	17.4%	2	5.7%	0	0.0%	
**Marital status**													p < 0.001
Single	1,000	41.0%	29	51.8%	45	42.5%	75	48.4%	12	34.3%	2	40.0%	
Married/ Civil partnership	1,439	59.0%	27	48.2%	61	57.6%	80	51.6%	23	65.7%	3	60.0%	
**Ethnicity**													p < 0.001
White British	2,207	90.5%	49	87.5%	96	90.6%	142	91.6%	33	94.3%	5	100.0%	
Other ethnicities	232	9.5%	7	12.5%	10	9.4%	13	8.4%	2	5.7%	0	0.0%	
**Location**													p < 0.001
Urban area	2,115	86.7%	48	85.7%	91	85.9%	138	89.0%	29	82.9%	5	100.0%	
Rural area	324	13.3%	8	14.3%	15	14.2%	17	11.0%	6	17.1%	0	0.0%	
**Religion**													p < 0.001
No religion	1,797	73.7%	48	85.7%	84	79.3%	131	84.5%	30	85.7%	3	60.0%	
Have religion	642	26.3%	8	14.3%	22	20.8%	24	15.5%	5	14.3%	2	40.0%	
**Education**													p < 0.001
< 19 years	1,757	72.0%	43	76.8%	81	76.4%	127	81.9%	32	91.4%	3	60.0%	
≥ 19 years	682	28.0%	13	23.2%	25	23.6%	28	18.1%	3	8.6%	2	40.0%	
**Diabetes**													p < 0.001
No	2,338	95.9%	49	87.5%	92	86.8%	139	89.7%	28	80.0%	3	60.0%	
Yes	101	4.1%	7	12.5%	14	13.2%	16	10.3%	7	20.0%	2	40.0%	
**Hypertension**													p < 0.001
No	1,499	71.6%	26	52.0%	41	44.6%	65	44.5%	9	29.0%	0	0.0%	
Yes	595	28.4%	24	48.0%	51	55.4%	81	55.5%	22	71.0%	3	100.0%	

**Fig 1:**
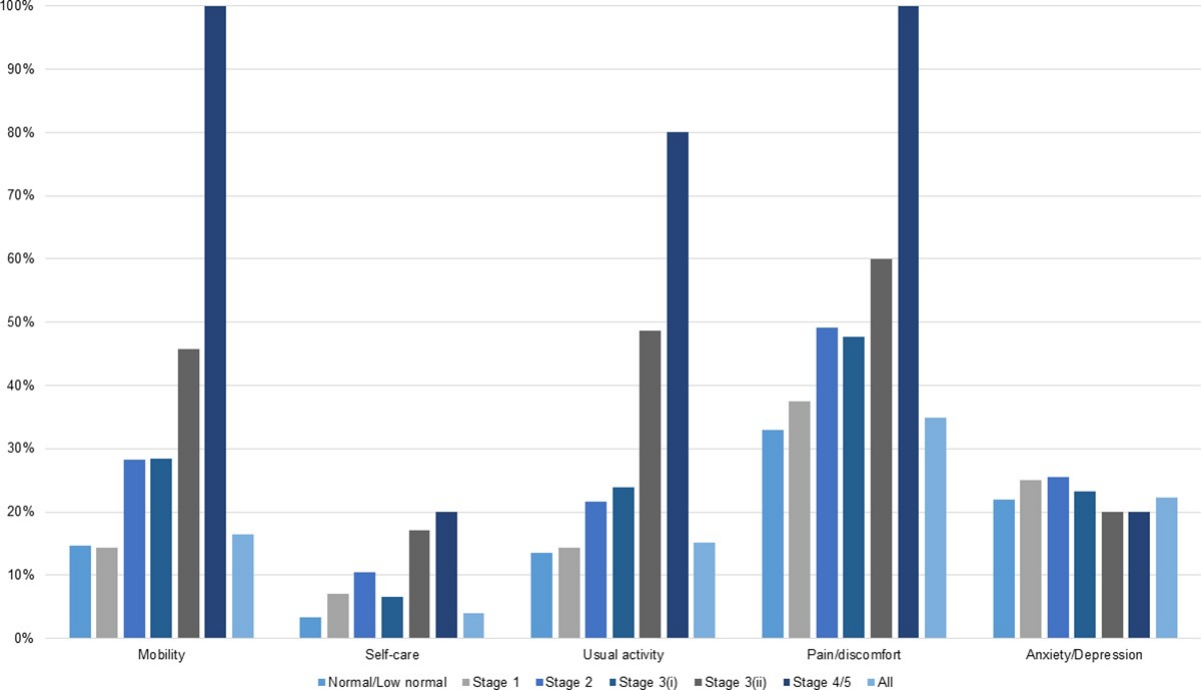
The proportion of respondents reporting any problems in each domain of EQ-5D questionnaire. Note: Stage 3(i): Stage 3 CKD with albuminuria, Stage 3(ii): Stage 3 CKD without albuminuria.

The proportions of respondents reporting any problems to the EQ-5D questionnaire are presented in Fig 1. In general, respondents seemed to report more problems in pain/discomfort and anxiety/depression domains than in usual activity, mobility and self-care domains. The proportion of those answering “No problems” was 83.6%, 96%, 84.9%, 65% and 77.8% in mobility, self-care, usual activity, pain/discomfort and anxiety/depression, respectively. In all stages of CKD, the pain/discomfort domain captured the highest proportion of people reporting the second level in three levels of EQ-5D-3L questionnaire, which were 30.3%, 32.1%, 43.4%, 43.2% and 51.4% in normal/low normal eGFR, stage 1, stage 2, stage 3 without albuminuria, stage 3 with albuminuria and stage 4/5 CKD, respectively. Fewer than 8.6% of the respondents reported ‘‘Extreme problem” for all dimensions.

**Fig 2:**
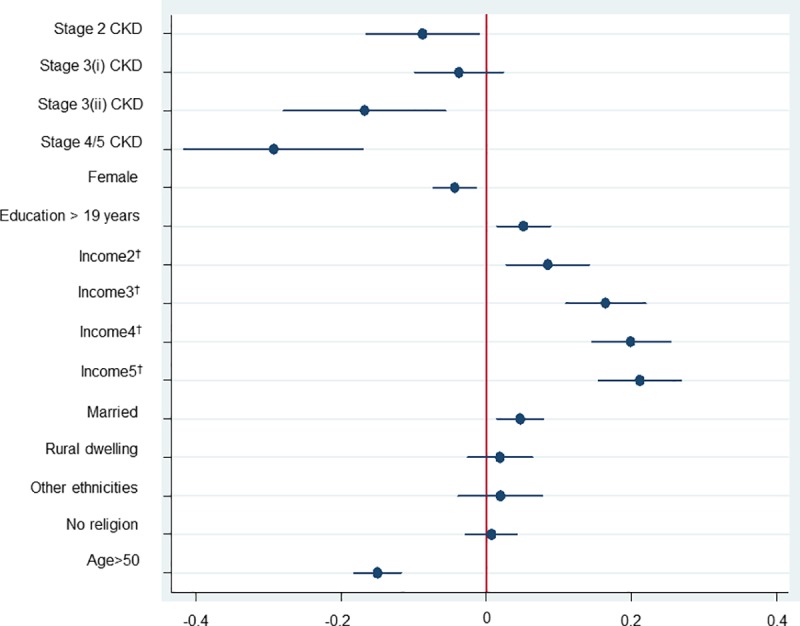
Multivariable Tobit model exploring the relationship between CKD severity and HRQoL (the base case model). Note: †: Income2 (>£11,142.86 & ≤£19,090.91), Income3 (>£19,090.91 & ≤£29,166.67), Income4 (>£29,166.67 & ≤£45,138.89), Income5 (>£45,138.89); (i) Stage 3 CKD without albuminuria, (ii) Stage 3 CKD with albuminuria. Bar represents 95% Confidence Intervals (CI), Dot represents the coefficient of the multivariable Tobit model.

In the base case model, a significant decrement of 0.11, 0.18 and 0.28 in health utility index was experienced by individuals with stage 2, stage 3 with albuminuria and stage 4/5 CKD compared to those with normal/low normal kidney function and stage 1 CKD, after adjustment for the other covariates. For sociodemographic data, people who were married and had more than 19 years of education had a better QoL than the comparator groups. Higher income was associated with better reported QoL. Compared to the lowest equivalised income quintiles (*≤*£11,142.86 per year), people with Second lowest Quintile (>£11,142.86 & *≤*£19,090.91), Middle Quintile (>£19,090.91 & *≤*£29,166.67), Second highest Quintile (>£29,166.67 & *≤*£45,138.89) and Highest Quintile (>£45,138.89) had significantly higher health utility indices of 0.08, 0.16, 0.20, 0.21, respectively. Females and people aged over 50 had worse health status with a decrement of 0.05 and 0.16 compared to male and those under 50 years old (Fig 2).

### Sensitivity analysis

The results of the sensitivity analyses are shown in supplementary document (S1 Table). When repeating the analysis using three sets of possible endogenous comorbidities (diabetes; hypertension; diabetes and hypertension), CKD continued to have a significant independent impact on HRQoL though its effect was attenuated and the coefficient of stage 4/5 CKD was no longer significant in the model including both diabetes and hypertension (-0.212, 95% CI: -0.451, 0.027). In the saturated models, CKD status continued to significantly and independently predict HRQoL though again its effect was attenuated. (S1 Fig and S2 Fig in the supplement present these results.)

### Exploring the relationship between CKD status and specific domains of health in EQ-5D

In Table 2, the results of the ordered logistic regressions examining the relationships between specific domains of health and CKD stage are presented in the form of estimated average marginal effects. As the coefficients in nonlinear models are not intuitively meaningful, marginal effects are another popular means to present results [25]. In brief while the presence and severity of CKD was related to problems with mobility, usual activity and pain/discomfort, no significant relationship was found in the self-care and anxiety/depression domains. Patients in stage 4/5 CKD, were respectively 59.5%, 38.7%, 36.3% points less likely to report “no problems” in mobility, usual activity and pain/discomfort compared to those with normal/low normal kidney function and stage 1 CKD. However, there were significant 52.1%, 27.4%, 25.4% points increases in the probability of reporting “some problems” in these three domains. The probabilities of reporting “extreme problems” was 7.4% and 10.9% points in mobility and pain/discomfort domains. Similarly, individuals with stage 3 CKD with albuminuria were less likely to report “no problems” and more likely to report “some problems” in mobility and usual activity. For individuals with less severe kidney disease, respondents with stage 2 CKD were also less likely to report “no problems” and more likely to report “some problems” or “extreme problems” in mobility and pain/discomfort domain of health.

**Table 2:** Estimated marginal effects.

EQ-5D domain	Stage 2 CKD	Stage 3 CKD (without albuminuria)	Stage 3 CKD (with albuminuria)	Stage 4/5 CKD
**Mobility**				
No problems	-0.070*	-0.063	-0.180*	-0.595**
Some problems	0.069*	0.061	0.175*	0.521**
Confined to bed	0.001	0.001	0.005	0.074*
**Self-care**				
No problems	-0.033	-0.006	-0.066	-0.097
Some problems	0.031	0.006	0.062	0.090
Unable to wash/dress	0.002	0.000	0.004	0.007
**Usual activity**				
No problems	-0.036	-0.048	-0.183*	-0.387*
Some problems	0.031	0.041	0.148*	0.274**
Unable to perform usual activity	0.005	0.007	0.035*	0.112
**Pain/discomfort**				
No pain or discomfort	-0.115*	-0.076	-0.135	-0.363**
Moderate pain or discomfort	0.096*	0.064	0.111	0.254**
Extreme pain or discomfort	0.019*	0.012	0.024	0.109**
**Anxiety/Depression**				
Not anxious or depressed	-0.041	-0.002	-0.012	0.041
Moderately anxious or depressed	0.035	0.002	0.011	-0.036
Extremely anxious or depressed	0.006	0.000	0.002	-0.005

The dependent variables (Mobility, Self-care, Usual activity, Pain/discomfort, Anxiety/Depression) are ordered variables taking values of no problems, some problems and extreme problems. We used the ordered Probit model, adjusted for equivalised household income, age, gender, marital status, education level, ethnicity, religion and location. All models were appropriately weighted for the sample.

*Denotes significant at 5%

**Denotes significant at 1%.

### The burden of chronic kidney disease and projections among those with diabetes to 2025

**Fig 3:**
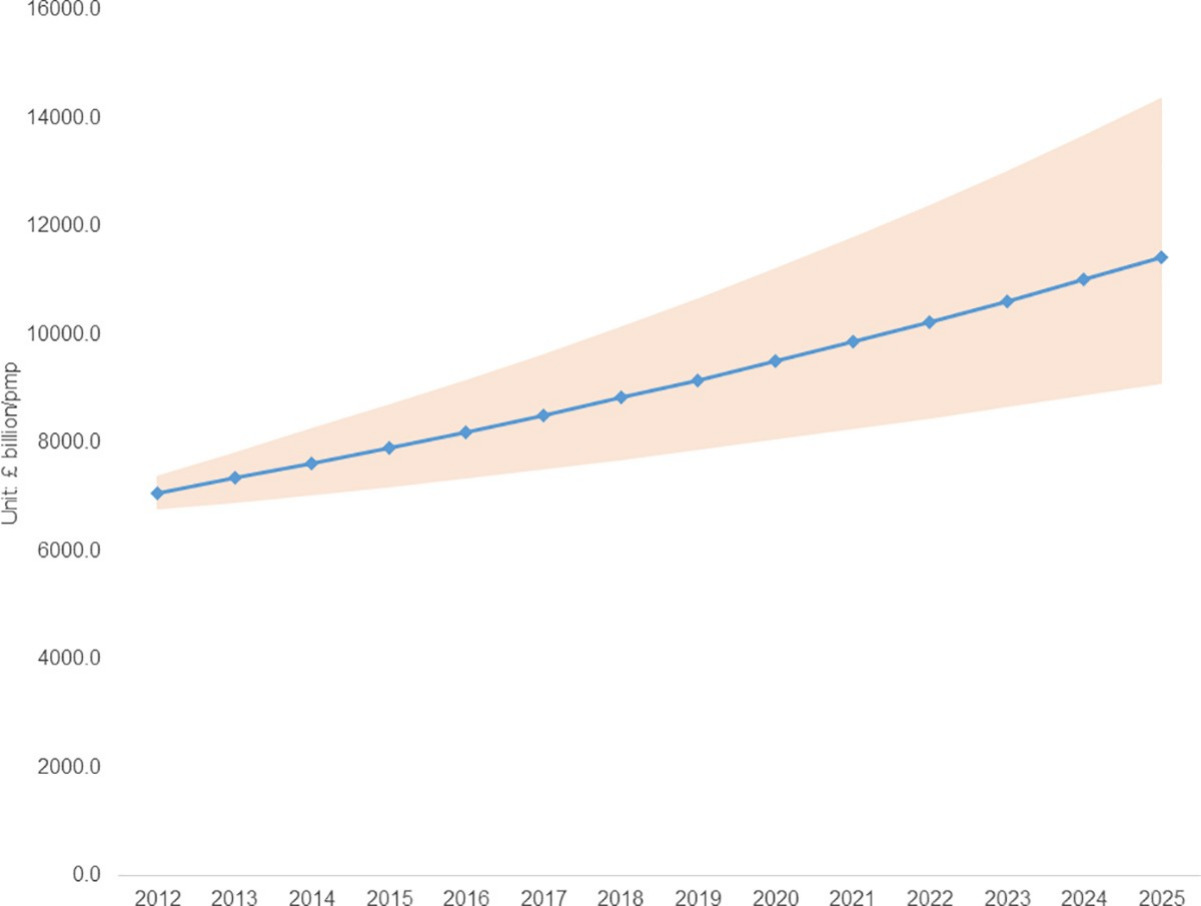
Projected economic burden of stages 3–5 CKD from 2012 up to 2025 in the UK. pmp: per million diabetic population. Line represents the prediction economic burden per million diabetic population from 2012 up to 2025; the dark area represents 95% Confidence Intervals (CI).

Using the base case model (Model 1) to project the economic burden of CKD to 2025, the QALY decrement between patients with CKD and individuals from a healthy population at the same age showed significant differences. In males, those with stage 2, stage 3 with albuminuria and stage 4/5 CKD suffered a loss of 3.5, 2.8 and 4.2 QALY, respectively. In females, stage 2, stage 3 with albuminuria and stage 4/5 CKD are associated with a loss of 4.8, 2.3 and 6.8 QALY, respectively. Applying a reimbursement threshold of £30,000 per QALY in the UK setting, the monetised burden of reduced HRQoL due to stage 2, stage 3 with albuminuria and stage 4/5 CKD were £103,734; £83,399; £125,335 in males and £143,582; £70,288; £203,804 in females, respectively. Extrapolating these out to 2025, per million of persons with diabetes in the UK, the present value of QALYs lost associated with CKD stages 3–5 will be approximately £11.4 billion (95% CI: 9.1, 14.4) of which 55% will be in women and 45% will be in men (Fig 3). The allocated proportion for stage 3, stage 4 and stage 5 CKD will be 79%, 15% and 6% of the total burden. Full details in the breakdown by gender and stage are presented in the supplement (S3 Fig).

## Discussion

Our study shows a clear decrement in HRQoL associated with CKD. The finding is consistent with previous studies that focused on those with established disease regardless of the instruments used to measure QoL [8–12,17,26]. After adjustment for other covariates in the ordered Probit models, mobility, usual activity and pain/discomfort were three dimensions of health that were more likely related to CKD; in which pain/discomfort showed a more significant marginal effect on the HRQoL. This finding is consistent with a study from Lee et al. (2012) in which mobility and pain/discomfort were the domains largely affected [8]. Unfortunately, chronic pain has not been considered a major problem in individuals with CKD to date [27]. We found that, consistent with other studies, having a higher income [8], being married [8,16], having better education [8,16], being younger [8,11] and male [8–11] were all associated with higher levels of HRQoL. Conversely, Yang et al. (2015) reported that older patients had better QoL than younger patients which might be due to the older sample used in that study (aged 60 and over) and the potential adaptation to chronic conditions and adjustment of expectations [14].

Our illustrative estimate underscores the magnitude of the HRQoL burden associated with CKD when expressed in monetary terms. Per person the lifetime HRQoL of CKD was £103,734; £83,399; £125,335 in males and £143,582; £70,288; £203,804 in females with stage 2, stage 3 with albuminuria and stage 4/5 CKD, respectively. Extrapolating these figures to reflect the growing prevalence of diabetes and CKD among individuals with diabetes further highlights the magnitude of the issue. Extrapolating the lifetime monetised value to the population the lost HRQoL is equivalent to £7.18 billion (£3.18 billion for males and £3.90 billion for females) in 2012, a figure we have shown seems destined to increase with the prevalence of diabetes in the short to medium term. Despite the significant burden in terms of HRQoL, CKD continues to be allocated a meagre share of research resources according to the budget expenditure report of the Centers for Disease Control and Prevention [28]. In addition, CKD awareness remains extremely low in both high-income and low and middle-income countries [29], this lack of awareness undermining the development of a more appropriate policy response in terms of the provision for services, the promotion of effective prevention and education services, and the priority afforded research in this area. Based on our findings clearly a reappraisal of the priority attached CKD is warranted.

### Strengths

This is the first population-based analysis of the relationship between CKD and HRQoL using the preference based EQ-5D utility in the UK. While most of other studies focused on moderate to severe CKD or ESRD, our study investigated the HRQoL in all stages of CKD.

This is also the first study to monetise the QALY loss due to kidney impairment in each CKD stage, whilst controlling for a range of covariates, and extrapolates this value to forecast the burden for DKD up to 2025.

In order to capture the censored nature of health utility index, we applied a set of multivariable Tobit models as this type of model has been widely used in this specific type of data and is better suited to the analysis of censored data than ordinary least squares [20]. We also investigated the impact of hypertension and diabetes as potential endogenous regressors of CKD in explaining the decrement in health utility.

### Limitations

We used HSE 2010 data, which though the most recent year for which data is available is now some 8 years out of date. We use cross sectional data that examines associations rather than causal relationships, again though in the absence of large follow-up studies this is a restriction imposed on us. This is similarly the case with respect to the use of EQ-5D-3L which is known to be less sensitive and to be more likely to suffer ceiling effects compared to the 5L or disease specific QoL measures. This again however, was a restriction imposed on us by the data.

There is also the uncertainty in the estimated economic burden as this work was based on another study to obtain the projected prevalence of CKD per million diabetes population up to 2025. However, we obtained the 95% CI of the estimates from a study of Kainz et al. whilst still recognising the inherent uncertainty in any kind of projection [24]. This is similarly the case with the use of estimated life expectancy.

There are also pitfalls in methods to estimate eGFR and classify CKD. In HSE 2010, CKD was classified using the estimated glomerular filtration measures (eGFR) obtained from MDRD equation [5,30]. While the CKD-EPI equation could provide more accurate estimates than MDRD equation, it was also reported that the global prevalence of CKD may have been overestimated by >50% due to the cumulative impact of pitfalls in prevalence estimates based on these two equations [31]. Moreover, false positive test results while screening and monitoring CKD has been a common issue and could affect the QoL of patients [32]. However, this issue is related to the standard technique and equation to estimate eGFR value and need further studies to address.

The small number of observations in persons with stage 4/5 CKD will inevitably increase uncertainty around estimates for more severe disease. However, as more severe disease is less prevalent in the population, this is an inevitable compromise associated with the use of a population-based survey. Only by increasing substantially the sample size–a matter beyond our control—could this be addressed.

## Conclusion

The severity of CKD predicts self-reported HRQoL. We found that as CKD severity increased so the HRQoL fell in a manner consistent with intuition. Using estimated life expectancy, CKD prevalence forecasts and the reimbursement threshold used in the UK for the valuation of QALYs, we estimated the HRQoL burden of CKD per million of the population with diabetes to rise from £7.08 billion to £11.4 billion between 2012 and 2025 in the UK.

## Supporting information

S1 Fig The marginal effects on HRQoL from Model 2. (TIF)

S2 Fig The marginal effects on HRQoL from Model 3. (TIF)

S3 Fig Projected economic burden of CKD up to 2025 in the UK by stage and gender. (TIF)

S1 Table Sensitivity analysis results. (DOCX)
