# Supplementary figures and images for: Chronic kidney disease, health-related quality of life and their associated economic burden among a nationally representative sample of community dwelling adults in England
Source: PLoS One. 2018 Nov 26;13(11):e0207960. doi: 10.1371/journal.pone.0207960 (PMC6258125; doi:10.1371/journal.pone.0207960)

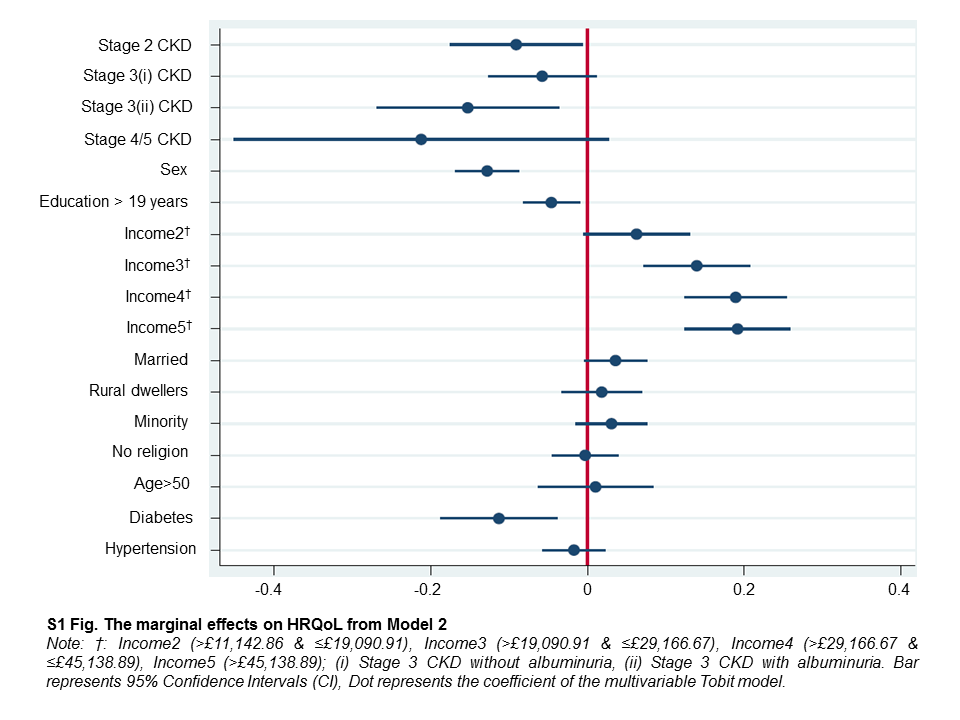

Supplement: S1 Fig — (TIF) [file pone.0207960.s001.tif]

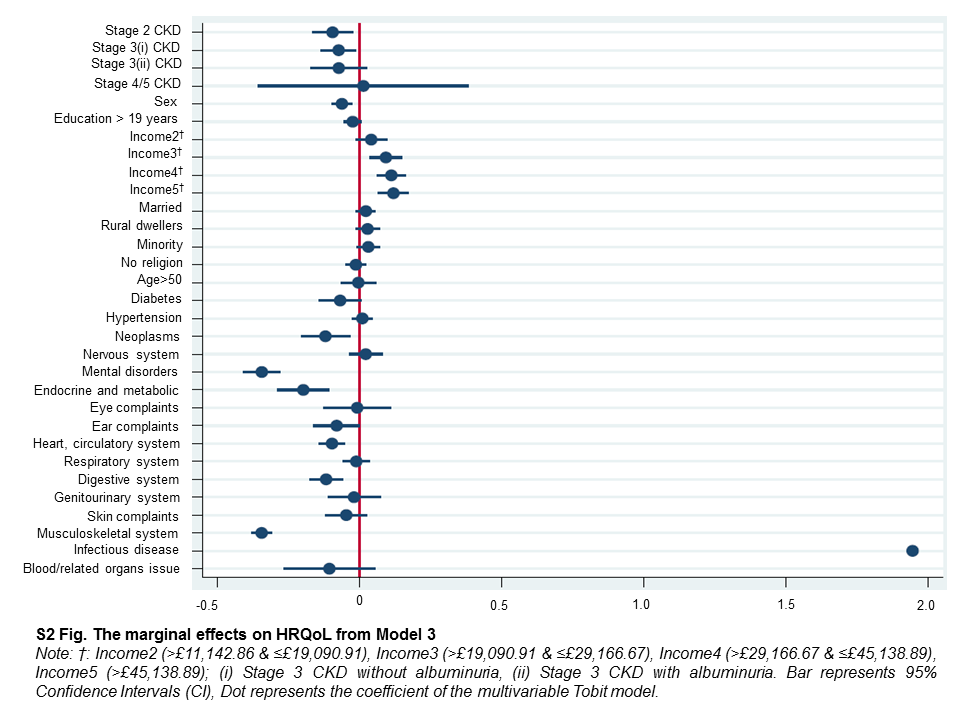

Supplement: S2 Fig — (TIF) [file pone.0207960.s002.tif]

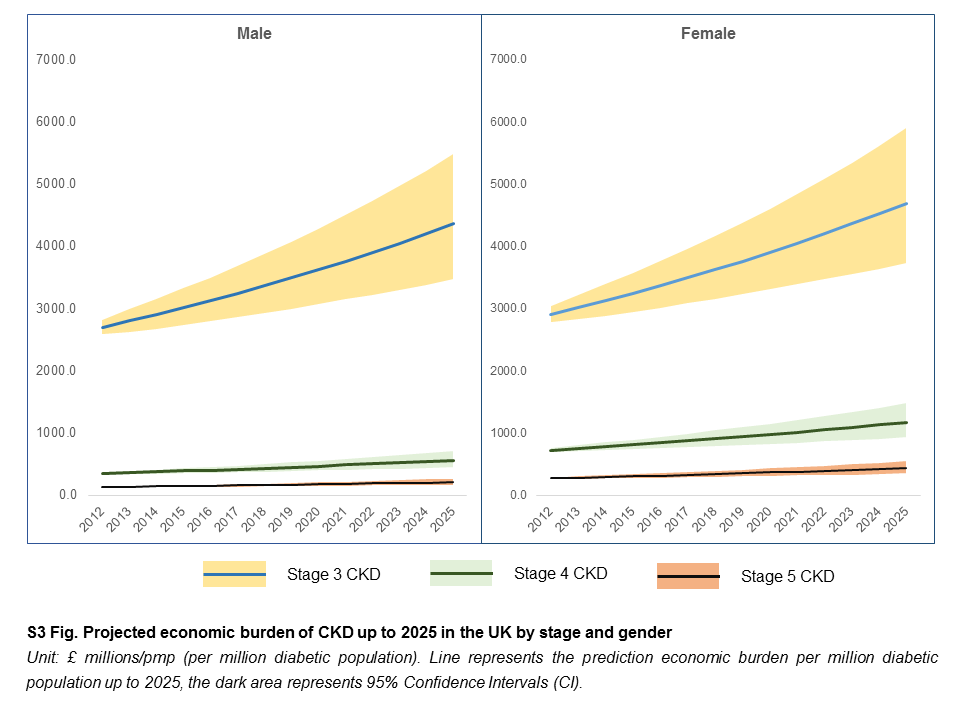

Supplement: S3 Fig — (TIF) [file pone.0207960.s003.tif]
