# Supplementary material for: Chronic kidney disease, health-related quality of life and their associated economic burden among a nationally representative sample of community dwelling adults in England
Source: PLoS One. 2018 Nov 26;13(11):e0207960. doi: 10.1371/journal.pone.0207960 (PMC6258125; doi:10.1371/journal.pone.0207960)
Supplement: S1 Table — (DOCX) [file pone.0207960.s004.docx]

**S1 Table. Sensitivity analysis results**

|  | **Weighted model ^a^** | | | **Unweighted model ^b^** | | |
| --- | --- | --- | --- | --- | --- | --- |
|  | **Health utility decrement** | **95% CI** | | **Health utility decrement** | **95% CI** | |
| ***Health utility decrement in each CKD stage ^c^ (Model 1)*** | | | | | | |
| **Stage 2 CKD** | -0.112* | -0.189 | -0.034 | -0.088* | -0.167 | -0.010 |
| **Stage 3 CKD**  **(without albuminuria)** | -0.062 | -0.128 | 0.005 | -0.037 | -0.099 | 0.024 |
| **Stage 3 CKD**  **(with albuminuria)** | -0.185* | -0.299 | -0.071 | -0.168* | -0.280 | -0.056 |
| **Stage 4/5 CKD** | -0.284^**^ | -0.408 | -0.160 | -0.293^**^ | -0.417 | -0.170 |
| ***Health utility decrement in each CKD stage, exploring the impact of diabetes ^c^*** | | | | | | |
| **Stage 2 CKD** | -0.104* | -0.181 | -0.026 | -0.081* | -0.159 | -0.003 |
| **Stage 3 CKD**  **(without albuminuria)** | -0.059 | -0.126 | 0.008 | -0.034 | -0.096 | 0.028 |
| **Stage 3 CKD**  **(with albuminuria)** | -0.175* | -0.292 | -0.059 | -0.156* | -0.271 | -0.040 |
| **Stage 4/5 CKD** | -0.247* | -0.404 | -0.091 | -0.261* | -0.409 | -0.113 |
| ***Health utility decrement in each CKD stage, exploring the impact of hypertension ^c^*** | | | | | | |
| **Stage 2 CKD** | -0.099* | -0.183 | -0.014 | -0.077 | -0.163 | 0.009 |
| **Stage 3 CKD**  **(without albuminuria)** | -0.058 | -0.127 | 0.011 | -0.033 | -0.097 | 0.030 |
| **Stage 3 CKD**  **(with albuminuria)** | -0.155* | -0.270 | -0.040 | -0.139* | -0.256 | -0.023 |
| **Stage 4/5 CKD** | -0.239* | -0.422 | -0.056 | -0.244* | -0.433 | -0.056 |
| ***Health utility decrement in each CKD stage, exploring the impact of diabetes and hypertension ^c^ (Model 2)*** | | | | | | |
| **Stage 2 CKD** | -0.091* | -0.175 | -0.007 | -0.077 | -0.163 | 0.009 |
| **Stage 3 CKD**  **(without albuminuria)** | -0.058 | -0.127 | 0.011 | -0.033 | -0.097 | 0.030 |
| **Stage 3 CKD**  **(with albuminuria)** | -0.153* | -0.269 | -0.037 | -0.139* | -0.256 | -0.023 |
| **Stage 4/5 CKD** | -0.212 | -0.451 | 0.027 | -0.244* | -0.433 | -0.056 |
| ***Health utility decrement in each CKD stage, exploring the impact of other health issues ^d^ (Model 3)*** | | | | | | |
| **Stage 2 CKD** | -0.094* | -0.166 | -0.022 | -0.078* | -0.151 | -0.004 |
| **Stage 3 CKD**  **(without albuminuria)** | -0.073* | -0.134 | -0.011 | -0.043 | -0.099 | 0.013 |
| **Stage 3 CKD**  **(with albuminuria)** | -0.072 | -0.171 | 0.027 | -0.068 | -0.167 | 0.031 |
| **Stage 4/5 CKD** | 0.014 | -0.355 | 0.384 | -0.006 | -0.351 | 0.339 |

*(a) Weighted for the nurse-based measures in HSE 2010; (b) Unweighted for the nurse-based measures in HSE 2010; (c) Multivariable model contains equivalised household income, age, gender, marital status, education level, ethnicity, religion and location; (d) Multivariable model contains equivalised household income, age, gender, marital status, education level, ethnicity, religion and location, diabetes, hypertension and a range of other health issues including neoplasms, endocrine and metabolic, mental disorders, nervous system, eye complaints, ear complaints, heart and circulatory system, respiratory system, digestive system, genitourinary system, skin complaints, musculoskeletal system, infectious disease and blood and related organs issue.*

** Denotes significant at 5%; ** Denotes significant at 1%, CI – Confidence intervals.*
